# Looking While Unhappy: A Mood-Congruent Attention Bias Toward Sad Adult Faces in Children

**DOI:** 10.3389/fpsyg.2018.02577

**Published:** 2018-12-18

**Authors:** Nicola Grossheinrich, Christine Firk, Martin Schulte-Rüther, Andreas von Leupoldt, Kerstin Konrad, Lynn Huestegge

**Affiliations:** ^1^Child Neuropsychology Section, Department of Child and Adolescent Psychiatry and Psychotherapy, University Hospital of the RWTH Aachen, Aachen, Germany; ^2^Neurophysiological Section, Department of Child and Adolescent Psychiatry, Psychosomatics, and Psychotherapy, Medical Faculty, University of Cologne, Cologne, Germany; ^3^Department of Social Sciences, Institute of Health Research and Social Psychiatry, Catholic University of Applied Sciences of North Rhine – Westphalia, Cologne, Germany; ^4^Translational Brain Medicine in Psychiatry and Neurology, Department of Child and Adolescent Psychiatry, Psychosomatics, and Psychotherapy, JARA Brain Translational Medicine, University Hospital of the RWTH Aachen, Jülich, Germany; ^5^Health Psychology, University of Leuven, Leuven, Belgium; ^6^JARA-Brain Institute II Molecular Neuroscience and Neuroimaging, Research Centre Jülich, Jülich, Germany; ^7^Institute of Psychology, University of Würzburg, Würzburg, Germany; ^8^Institute of Psychology, RWTH Aachen University, Aachen, Germany

**Keywords:** eye tracking, emotion regulation, mood induction, attention bias, major depression, adaptive role

## Abstract

A negative mood-congruent attention bias has been consistently observed, for example, in clinical studies on major depression. This bias is assumed to be dysfunctional in that it supports maintaining a sad mood, whereas a potentially adaptive role has largely been neglected. Previous experiments involving sad mood induction techniques found a negative mood-congruent attention bias specifically for young individuals, explained by an adaptive need for information transfer in the service of mood regulation. In the present study we investigated the attentional bias in typically developing children (aged 6–12 years) when happy and sad moods were induced. Crucially, we manipulated the age (adult vs. child) of the displayed pairs of facial expressions depicting sadness, anger, fear and happiness. The results indicate that sad children indeed exhibited a mood specific attention bias toward sad facial expressions. Additionally, this bias was more pronounced for adult faces. Results are discussed in the context of an information gain which should be stronger when looking at adult faces due to their more expansive life experience. These findings bear implications for both research methods and future interventions.

## Introduction

A mood-congruent attention bias (defined as a biased deployment of attention toward mood-congruent information), is assumed to maintain a sad mood, as postulated, for example, in the theoretical framework of the development and maintenance of major depressive disorder (MDD, Clasen et al., 2012). According to Beck’s schema theory of depression (e.g., Beck, 1967, 1976), negative schemas are presumed to be activated through negative stressors, which in turn affect thoughts and judgments about the self, the world, and the future (usually referred to as Beck’s cognitive triad). According to this model, depressed individuals attend to information that is congruent with their negative cognitive schemas. Though Beck’s model is rather descriptive in nature (Haaga et al., 1991), the concept of a negative bias in social cognition, for the development of MDD, has influenced the scientific debate since the 80s (Teasdale, 1983). Specifically, some researchers and clinicians assume that the negative information processing bias represents a stable trait, which precedes and causes negative moods and thoughts and eventually leads to depression (Fritzsche et al., 2010). In contrast, others refer to the negative bias as a transient state in that it co-occurs with MDD (Lewinsohn et al., 1981) as a consequence of sad mood and fades away when MDD (and the inherent sad mood) is remitted. In the context of this scientific debate, both clinical studies and experiments with healthy individuals were conducted, the latter typically involving mood induction techniques to enhance our understanding of the functional role and underlying mechanisms of the mood-congruent attention bias.

While there is evidence for very early (pre-conscious) mood-congruent attention processing in anxiety (e.g., Mogg et al., 1994, 1995, 2017; Okon-Singer, 2018), the results regarding sad/depressive mood-congruent biases were rather mixed: While some studies report mood-congruent biases in depression and non-clinical dysphoria (Gotlib and McCann, 1984; Gotlib and Cane, 1987; Unruh et al., 2018), others failed to observe such a bias (Mogg et al., 1993; Bradley et al., 1995; Cheng et al., 2015).

In order to shed more light on such heterogeneous results, Bradley et al. (1997) investigated the temporal characteristics of the mood-congruent attention bias, using a dot probe task. As a consistent result the mood-congruent bias for a sad mood was most prominent for long lasting exposure durations (>1 s), while the mood-congruent bias for anxiety – as reported in former studies – was rather associated with short (pre-conscious) exposure. Thus, in contrast to anxiety, a sad mood is not associated with an initial orienting attention bias. Instead, once mood-congruent information is in the focus of attention, a sad individual may have greater difficulty to disengage their attention from corresponding material.

Temporal characteristics therefore, appear to be essential to investigate attentional biases. Note, however, that behavioral tasks such as the dot-probe task are criticized due to their poor psychometric characteristics, which might be attributed to the indirect inference of attentional biases using behavioral responses (for an overview see Zvielli et al., 2015). Instead, eye-tracking approaches seem to guarantee a more direct and improved access to determine attentional biases.

A study utilizing eye-tracking to accurately assess visual attention biases, reported that a negative mood-congruent attention bias was only present in younger adults (≤25 years), but not in older (≥58 years) adults (Isaacowitz et al., 2008). The authors explained their findings by referring to “socioemotional selectivity theory”: When the time horizon shrinks (e.g., for elderly people), individuals become increasingly selective in that they exhibit a general motivational shift in attention toward positive over negative information (Carstensen, 2006). For younger individuals the study -which only involved emotional faces of adults as stimulus material -assumes that the mood-congruent attention bias might serve to gain information about causes and consequences of a sad mood from more a life-experienced persons (Carstensen et al., 2006), suggesting an adaptive function of the mood-congruent attention bias. Note, however, that Isaacowitz et al. (2008) did not explicitly manipulate the age of the depicted facial expressions to directly address this issue.

Previous research suggests that face processing might indeed depend on the age relationship between the viewer and the to-be-looked-at person. For example, face processing is assumed to re-orientate from parents toward peers in puberty (similarity approach, Picci and Scherf, 2016). Scherf and Scott (2012) explained this finding by postulating specific developmental tasks (in terms of general problems to be solved at certain stages of development) as essential in the context of face processing. One implication is that young children are not likely to exhibit an “own-age” bias in face processing if the developmental task for an individual is to discriminate caregiver(s) from others. According to the theoretical framework of Scherf and Scott (2012), we assume that attentional biases in children are related to the attachment behavioral system, initially postulated by Bowlby (1969) as an inborn strategy for emotion regulation. Although the attachment system is most critical during the early years of life, Bowlby assumed that it is active over the entire life span (Bowlby, 1988). Three main coping strategies comprise acknowledgment/display of distress, support seeking and engagement in instrumental problem solving (Waters et al., 1998). An attentional bias toward adult sad faces in children can therefore be interpreted as support seeking behavior. Specifically, adaptive and maladaptive emotion regulation strategies to regulate a sad mood, might be taught by caregivers and have an effect on mood improvement or on mood worsening, respectively (Silk et al., 2003).

Until today, the mood-congruent attention bias is assumed to maintain the actual sad mood and dysphoria. As a consequence, the mood-congruent attention bias has predominantly been seen as maladaptive (Clasen et al., 2012), whereas a potentially adaptive aspect of this spontaneous behavior (as postulated in Isaacowitz et al., 2008 and implied by the attachment behavioral system postulated by Bowlby, 1969, see above) has largely been neglected until today.

In the present study, we built on the design by Isaacowitz et al. (2008) who investigated the attention bias (via eye tracking) of younger and elderly individuals, when a sad mood was induced. However, instead of manipulating the age of the participants, we selectively focused on a group of children and manipulated the age of the depicted faces. We hypothesized that the induction of a sad mood in children, should lead to a corresponding mood-congruent gaze bias, especially toward adult faces compared to faces depicting children.

## Materials and Methods

### Participants

20 children (aged 6;6 to 12;11 years, *M* = 9;6; *SD* = 1;10; 9 male) participated in the experiment. They were recruited by flyer distribution in German elementary schools and by local announcements. Only participants with normal vision were included. Based on a previously reported effect size for the three-way interaction of age group, mood induction and emotional facial expression in the somewhat related study by Isaacowitz et al. (2008), a power analysis (G^∗^Power, Faul et al., 2007; 1-beta = 0.80, alpha = 0.05) suggested a sample size of approximately 20 participants.

Temperaments were assessed using the temperament scales of the Junior Temperament and Character Inventory (JTCI 7-11R; Luby et al., 1999; Goth and Schmeck, 2009), which is based on a biosocial model postulated by Cloninger (1986). The biosocial model describes four dimensions of temperament, namely “harm avoidance,” “novelty seeking,” “reward dependence,” and “persistence.” “Harm avoidance” (characterized by worrying, pessimism and shyness) is a well-known risk factor for MDD, whereas the remaining three temperament dimensions were reported to be comparable in MDD and controls (Hansenne et al., 1999). Finally, behavioral problems were evaluated with the Child Behavior Checklist (CBCL/4-18, Achenbach, 1991; Arbeitsgruppe Deutsche Child Behavior Checklist, 1998) to control for depressive symptoms. The study was conducted in accordance with the Declaration of Helsinki and was approved by the local Ethics Committee. Written informed consent was obtained from the parents and their children. Children were paid for their participation.

### Mood Induction

Two short film clips were selected to induce a happy mood (“The Bare Necessities” of the “Jungle Book”) and a sad mood (“Death of Simba’s father” in “Lion King”). Both clips were comparable in terms of length (3 min), color, sound, picture motion, and complexity. The content of both clips was chosen to be understandable without further explanation: The mood induction procedure was previously validated in 299 children aged 6–12 years (von Leupoldt et al., 2007). Mood was assessed using the paper and pencil version of the Self-Assessment Manikin (SAM, Hodes et al., 1985) on a 9-point Likert scale. During mood assessment, children rated how pleasant/happy/amused (9) or unpleasant/unhappy/sad (1) they felt immediately before and after watching the films.

For the main eye tracking data analysis, the data from individuals were excluded when the negative mood induction clearly failed (post – pre >0: 3 males; rigid response behavior for all mood assessments: 1 male). The eye tracking data from 16 children (children aged 6;6 to 12;11 years, M = 9;7; SD = 1;11; 5 male) were then entered into the final analysis. Note that this reduction in sample size inevitably was in line with a slight reduction in statistical power (see Discussion).

### Eye-Tracking Paradigm

The EyeLink 1000 eye tracker (SR Research Ltd., Mississauga, ON, Canada) was used to measure the pupil and the corneal reflection of the right eye. A chin and forehead rest was placed in front of the display monitor to attain a stable position of the participants’ heads. Eye movements were measured with a sampling rate of 500 Hz. A 5-point calibration was applied, that covered the whole visual field in which the stimuli were presented. After data collection, oculomotor parameters were extracted using the Data Viewer software (SR Research).

In each experimental trial, children viewed a pair of faces on a screen depicting a neutral and an emotional facial expression, respectively. The emotional stimuli either displayed a sad, happy, angry or fearful facial expression. All four emotional facial expressions were presented twice (left position and right position) using pictures of eight different adults and eight different children (balanced with respect to gender). The manipulation of emotional facial expression, type of stimuli (8 children, 8 adults), and position of the emotional facial expression (left, right) resulted in 128 trials (4 x 16 x 2) altogether, which were presented in randomized order. Face stimuli were displayed for 4,000 ms followed by an animated fixation stimulus which was presented for 500 ms. The fixation stimulus depicted a cat running in place and was introduced to ensure that children’s gaze returned to the center of the screen without further instruction. The children were instructed to look thoroughly at the faces on the screen. After each block (consisting of ten trials), a calibration of the eye tracker was carried out. A test run was conducted prior to the experiment to familiarize the children with the calibration procedure. The duration of the main eye tracking experiment (without test runs and instructions) amounted to approximately 15 min.

### Facial Stimuli

The facial stimuli were taken from the standardized Radboud Faces Database (Langner et al., 2010) and depicted adults and children. The age of the depicted faces could be estimated as elementary school age for children (reflecting the age of the participants) and between 20 and 30 years for adults. Therefore, the age of adults could be approx. the age of participant’s parents, when children were born. Alternatively, it is the age of caregivers in institutional contexts.

The test run was executed using facial stimuli from the Pictures of Facial Affect database by Ekman and Friesen (1976) to avoid habituation effects by presenting a different set of facial expressions.

### Procedure

Children were accustomed to the eye tracking procedure and initial mood ratings were obtained. Then happy vs. negative mood induction was administered using the aforementioned standardized film clips (“Jungle Book” for happy mood induction, and “Lion King” for sad mood induction) within a repeated measurements design. Thus, half of the children experienced the happy mood condition prior to the sad mood induction condition, while the other half experienced the reversed order. The children were instructed to watch carefully and encouraged to engage with the films. Before and after mood induction, current mood ratings were provided as illustrated in the “Mood induction” section. Immediately after each mood induction procedure, the main eye tracking experiment was performed.

**FIGURE 1:**
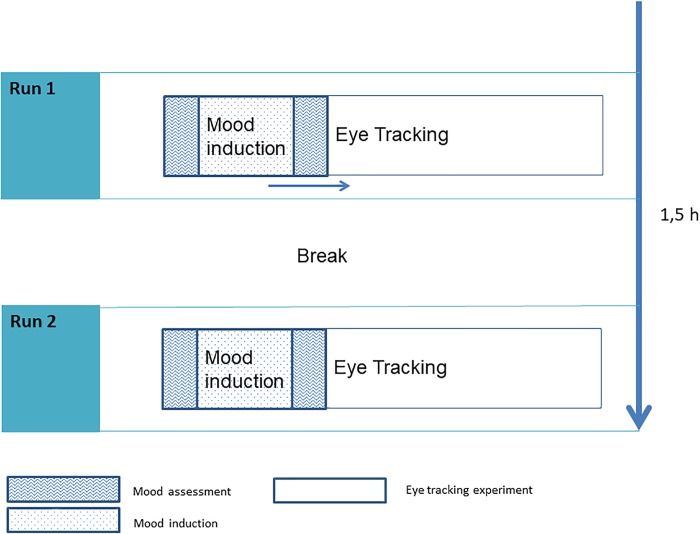
Experimental design.

Between both eye tracking experimental sessions (happy/sad session), all children were asked to run along a long floor and to color a mandala. We reasoned that activation (by running along the floor) and distraction (by coloring the mandala) would help to renew the initial mood before the next mood was induced. During the eye tracking experiment the mood was maintained by the test supervisor, through humming the song “The Bare Necessities” (“positive mood”) or by reminding the children of the sad mood induction content (“Think about the small lion who loses his father”) immediately before calibration. The whole experiment lasted approximately 1.5 h (see Figure 1).

### Statistical Analysis

The mood induction procedure was evaluated using a two-factorial ANOVA with two repeated measurements (valence: positive, negative; mood induction: pre, post). In line with previous studies (Isaacowitz et al., 2008), we first computed total fixation durations (fd), defined as the total time a subject looked at the emotional or neutral facial expression. Corresponding regions of interest (ROI) were equally sized for each face and trial. Then, a ratio score was computed as:

$$\mathrm{ratio}\;\mathrm{score}=({\mathrm{fd}}_{\mathrm{emotional}}-{\mathrm{fd}}_{\mathrm{neutral}})/({\mathrm{fd}}_{\mathrm{emotional}}+{\mathrm{fd}}_{\mathrm{neutral}}),$$

Indicating the viewer’s preference for either the emotional or the neutral facial expression during one trial. A positive score indicated a preference for the emotional facial expression, whereas a negative score indicated a preference for the neutral face (as in Isaacowitz et al., 2008). Eye movements were successfully tracked for at least 75% of all trials.

In order to further interpret the age-dependent mood congruent attentional bias (based on fixation duration ratios) against the backdrop of an initial orientation bias, we additionally examined the first view of the children using similar ratio scores (see the formula reported above). In detail, latency of the first view for each region of interest and the corresponding gaze duration were investigated separately.

For the main analysis, a three-factorial analysis of variance (ANOVA) was conducted on the ratio scores (mood induction: positive, negative; age of stimuli: children, adults; facial expression: sad, fearful, happy, angry) in a repeated measurements design. Contrasts between each negative facial expression and the positive facial expressions were performed, as neutral faces were presented in every trial for the purpose of the ratio score calculation. Each negative facial expression was analyzed separately, as the accuracy of identifying emotional facial expressions is known to increase in late childhood (particularly for neutral and sad facial expressions) while happy facial expressions seem to be identified easily (accuracy nearly 100%, Mancini et al., 2013). In addition, the main analysis was controlled by internal problem behavior, to exclude the possibility that depressive symptoms could explain our results, using an analysis of covariance (ANCOVA). When the assumption of sphericity was violated, Greenhouse-Geisser corrected values were reported. Additional correlational analyses were performed by calculating Pearson correlations, to investigate the relationship between the temperamental characteristics (which are associated with the development of stable mood states) of the participating children and the mood-dependent attentional biases.

## Results

### Mood Induction

**FIGURE 2:**
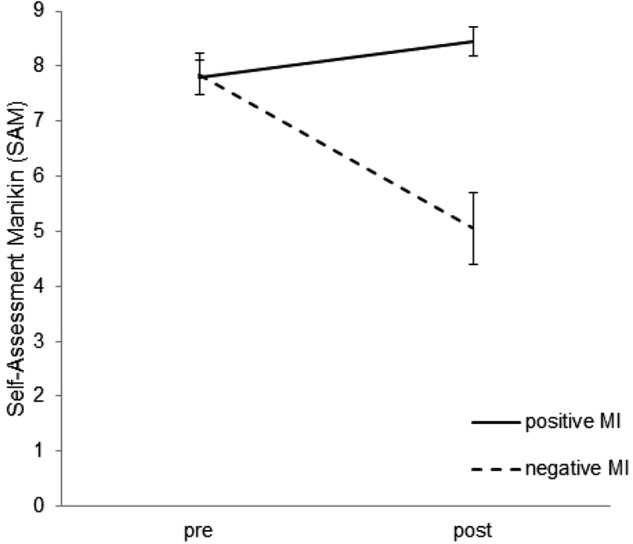
Mood prior and after the positive (happy) and negative (sad) mood induction (MI) procedure assessed with the Self-Assessment Manikin (SAM). Note that higher scores indicate better mood.

A two-factorial ANOVA yielded significant main effects of valence [positive/negative; *F (1,19)* = 13.47; *p* < 0.01, $\eta_{p}^{2}$ = 0.42] and mood induction [pre, post; *F (1,19)* = 10.68; *p* < 0.01, $\eta_{p}^{2}$ = 0.36] as well as a significant interaction [*F (1,19)* = 15.76; *p* < 0.01, $\eta_{p}^{2}$ = 0.45]. *Post hoc* one-sided contrasts revealed significant pre-post effects of both; the sad mood induction [*t(19)* = 3.99, *p* < 0.01] and the happy mood induction [*t(19)* = 2.04, *p* = 0.03, see Figure 2].

The initial mood and the actual mood before each mood induction did not differ [*F(2,18)* = 0.04; *p* = 0.96]. Additionally, the actual mood immediately before the sad mood induction and immediately before the happy mood induction was comparable [*F(1,19)* = 0.01; *p* = 0.92], suggesting that the activation/distraction measures were sufficiently effective.

### Eye Tracking Experiment

**FIGURE 3:**
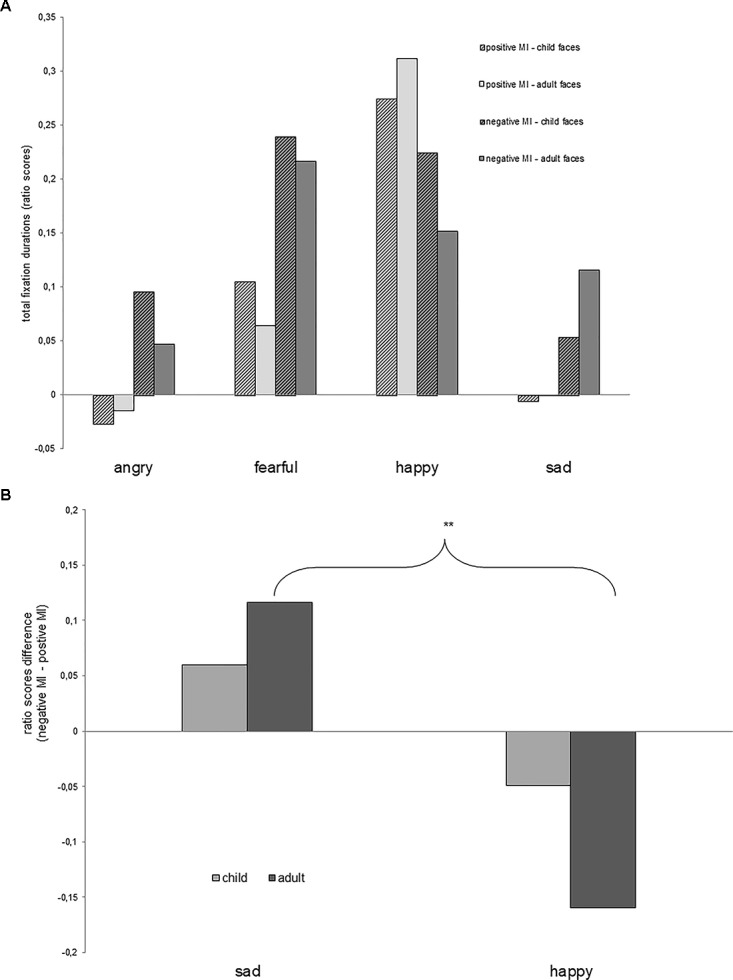
Figure **(A)** Ratio scores of total fixation durations as a function of depicted facial expression (fearful, angry, happy, and sad), type of stimuli (child and adult), and mood induction (MI: happy and sad). Note that positive ratio scores indicate a preference of the emotional (vs. neutral) facial expression. **(B)** Mood induction effect (in terms of ratio score differences) as a function of depicted facial expression (sad vs. happy) and age of stimuli (child vs. adult). ^∗∗^*p* < 0.01.

The attentional bias (based on total fixation durations, thereby reflecting rather long-lasting biases, see Figure 3) and the initial orientation (first view, representing short-lasting initial biases) were investigated.

#### Attentional Bias (Ratios Based on Total Fixation Duration)

The three-factorial ANOVA yielded a significant main effect of “facial expression” [*F(1.17,45)* = 5.81, *p* < 0.05, $\eta_{p}^{2}$ = 0.28] on ratio scores. Contrasts comparing all individual negative facial expressions (angry, sad, and fearful) to the positive expressions (happy facial expressions) revealed a significant effect between angry and happy facial expressions [*F(1,15)* = 6.73, *p* < 0.05, $\eta_{p}^{2}$ = 0.31] and between sad and happy facial expressions [*F(1,15)* = 6.32, *p* < 0.05, $\eta_{p}^{2}$ = 0.3] – reflecting an overall enhanced attentional bias toward happy (as compared with the negative) facial expressions, while the difference between fearful and happy facial expressions was not statistically significant [*F(1,15)* = 1.04, *p* = 0.32, Figure 3].

Importantly, and in line with our hypothesis, an interaction between “mood induction (positive, negative),” “facial expression (fearful, angry, sad, happy),” and “age of stimuli (children, adults)” [*F(3,45)* = 2.82, *p* < 0.05, $\eta_{p}^{2}$ = 0.16] was observed. This three-way interaction indicates that mood induction had different effects depending on the type of stimuli. To break down this interaction, further tests were performed comparing each negative (angry, sad, and fearful) facial expression to happy facial expressions (still involving the factor sad vs. happy mood induction). These comparisons yielded significant corresponding interactions when sad facial expressions were compared to happy facial expressions [*F(1,15)* = 9.51, *p* < 0.01, $\eta_{p}^{2}$ = 0.39] and when fearful facial expressions were compared to happy facial expressions [*F(1,15)* = 4.8, *p* < 0.05, $\eta_{p}^{2}$ = 0.24], while the contrast between angry facial expressions and happy facial expressions did not reveal a significance [*F(1,15)* = 0.47, *p* = 0.5].

Only the sad facial expression contrast survived Bonferroni correction (*p_sad_* = 0.008 < *α_Bonferroni_* = 0.017). Thus, a sad mood induction (in contrast to the happy mood induction) selectively yielded an attention bias toward sad faces, which was only significant for adult faces in contrast to faces depicting children. An analysis of combined (adult plus child) sad facial expressions (in contrast to combined happy facial expressions) did not yield a significant effect [*t(15)* = 1.26, *p* = 0.23]. A look at the negative MI data only (two rightmost bars for each depicted emotion) in Figure 3A, shows that there was an increase of the ratio score (reflecting stronger emotional bias) when presenting adult (vs. child) faces, only in the case of a sad depicted mood.

This analysis is equivalent to the respective contrasts, comparing both stimulus age conditions (adult: *M* = 0.14, *SD* = 0.33 vs. child: *M* = 0.05, *SD* = 0.3); *(15)* = 9.51, *p* = < 0.01, $\eta_{p}^{2}$ = 0.39) for sad facial expressions (in contrast to the happy facial expressions) reflecting an enhanced attentional bias to adult (vs. child) sad facial expressions when a sad (vs. happy) mood was induced (see greater difference in the mood induction effect between sad and happy faces for depicted adults compared to children, Figure 3B). As already described above, this reflects a more pronounced mood-congruent attention bias for depicted adult (vs. child) faces.

When internalizing problem behavior and age were included as covariates and the order of mood induction was included as between-subject factor, the overall pattern of results remained unchanged [“facial expression”: *F(3,36)* = 5.54, *p* < 0.05; “mood” x “facial expression” x “age of stimuli”: *F(3, 36)* = 2.96, *p* < 0.05], while there were no significant effects of internalizing problem behavior, age, and the order of the mood induction (all *p_s_* > 0.1).

#### Initial Orientation (First View)

Analyzing the latencies of the first view, the three-factorial ANOVA yielded a significant main effect of facial expression [*F(3,45)* = 15.74, *p* < 0.01, $\eta_{p}^{2}$ = 0.51] on corresponding ratio scores. Contrasts comparing all individual negative facial expressions (angry, sad, and fearful) to the positive expressions (happy facial expressions), revealed a significant difference between happy and sad facial expressions [*F(1,15)* = 36.8, *p* < 0.01, $\eta_{p}^{2}$ = 0.71] and between angry and happy facial expressions [*F(1,15)* = 21.8, *p* < 0.01, $\eta_{p}^{2}$ = 0.59], while no difference was found for the contrast between fearful and happy faces [*F(1,15)* = 0.05, *p* > 0.8] – reflecting an overall strong initial orientation toward happy and fearful faces in contrast to the neutral facial expression (as reflected in the ratio scores). No main effect of “age of stimuli” and no interaction with “age of stimuli” were observed. The analysis of the duration of the first view resulted in a significant interaction of mood’ and “facial expression” [*F(3,45)* = 3.4, *p* < 0.03, $\eta_{p}^{2}$ = 0.59], reflecting particularly shorter first view durations for fearful facial expressions [*F(1,15)* = 4.6, *p* < 0.05, $\eta_{p}^{2}$ = 0.24] in contrast to happy facial expressions, while children were in a sad (as opposed to happy) mood. Again, no main effect of age of stimuli and no interaction with age of stimuli’ were observed.

### Children Problem Behavior, Temperament and Attentional Biases

Correlation analyses revealed that externalizing problem behavior (CBCL) was associated with novelty seeking (JTCI), [*r(14)* = 0.56, *p* < 0.05], whereas internalizing problem behavior (CBCL) was correlated with harm avoidance (JTCI), [*r(14)* = 0.52, *p* < 0.05].

With respect to the findings of our main analysis, relationship between the attention bias to sad/happy facial expressions and temperaments were calculated in an exploratory manner. Harm avoidance was associated with an attentional bias toward sad adult facial expressions [*r*(14) = 0.6, *p* < 0.05] whereas this relationship could not be observed for facial expressions depicting children [*r*(14) = 0.41, *p* = 0.11], when a sad mood was induced. No relationship between harm avoidance and happy facial expressions [adult: *r*(14) = -0.21, *p* = 0.43; child: *r*(14) = -0.35, *p* = 0.18] were observed in the sad mood condition. In addition, a trend was found between novelty seeking and the attention bias toward adult happy faces [*r*(14) = 0.43, *p* = 0.096] while the children were in a sad mood. All other relationships did not reach significance (*p* > 0.20).

## Discussion

The eye tracking study presented in this article examined the mood-congruent attentional bias in typically developing children. To the best of our knowledge, this is the first study utilizing facial emotional expressions depicting adults and children, in order to shed more light on the age-dependent significance of this bias in children.

A mood-congruent gaze bias (ratio based on total fixation durations) especially toward adult sad faces was observed when children were exposed to a sad film clip. In line with findings of Bradley et al. (1997), the age-dependent bias was only evident in parameters reflecting a rather long lasting exposure to facial stimuli, while no corresponding effect was found for the initial orientation (first view). These results were not influenced by children’s age, internalizing problem behavior, or by the sequence in which a mood was induced in the experiment. Harm avoidance was associated with an attentional bias toward adult sad faces, an effect that was only present when a sad mood (and not a happy mood) was induced.

The current findings confirmed our main hypothesis. Specifically, we expected that a sad mood induction in children would lead to a spontaneous mood-congruent attention bias especially toward adult faces. This prediction was based on the assumption of a potentially adaptive function of the mood-congruent bias, namely to gain information about the causes, consequences, and potential mood regulation options in real life (Carstensen et al., 2006). This information gain should be especially pronounced when adult faces are involved in the experiment, since adults should be considered more informative based on their greater life experience, which makes them a better caregiver than children.

Interestingly, the main result were in line with a caregiver bias of facial recognition in children (Picci and Scherf, 2016). In contrast to the caregiver bias in facial recognition experiments we investigated attentional biases, which might be related to the attachment system, initially postulated by Bowlby (1969). According to Bowlby, proximity seeking behavior to caregivers is part of an adaptive behavioral system (attachment behavioral system), which is activated by stressful experiences, especially in children. The sad mood induction procedure might have activated this attachment behavioral system toward caregivers (here: adult facial expressions) by presenting a film clip in which a lion cub (child) loses his father by death.

The interaction pattern regarding the attentional bias toward child and adult faces might have important implications for suitable parental behavior and therapeutic interventions. One could argue, for example, that when parents, teachers, and/or psychotherapists communicate their own feelings and emotion regulation strategies (validation, self-disclosure) when children are sad, they will help their protégées to implement their individual emotional regulation strategy, to overcome or accept their own sadness. Overall, the observed sad mood-congruent bias of children toward adult facial expressions, can be interpreted as an adaptive strategy to gain information from more life experienced (older) individuals.

Notably, a mood-specific attention bias during a sad mood was observed toward sad faces. This specificity of information processing to sad material was repeatedly documented by contrasting the results of MDD (sad mood) to other disorders (mostly social phobia for which fear is the most salient emotion). For example, in MDD patients, but not in patients suffering from social phobia, an attention bias for stimuli implying sadness, was found (Gotlib et al., 2004). In a similar vein an attentional bias for disorder-related words was reported in depressive females. Again, the described attentional bias found in depressive females was not observed in social phobic females (Rinck and Becker, 2005). Moreover, depressed participants (in contrast to social phobic and healthy participants) required more degrees of emotional intensity to correctly identify happy facial expressions, while they required less emotional intensity to identify sad in contrast to angry facial expressions (Joormann and Gotlib, 2006). According to former findings, the significant positive association between the attention bias toward sad adult faces (when sad mood was induced) and “harm avoidance,” a personality trait which is discussed as a trait marker for liability to MDD (Smith et al., 2005), underlines the potential generalizability of the mood specific information bias, to personality traits reported in MDD (Hansenne et al., 1999), though the functional mechanisms related to “harm avoidance” in typically developed children and in MDD might differ. Nevertheless, at a developmental perspective, where sad children exhibit an attentional bias toward sad adult faces (which is true especially for harm avoidant children), our results might be the first clue to better understand the developmental mechanism of a maintained sad mood. In detail, any caregiver bias might be adaptive (when caregivers teach adaptive emotion regulation strategies) or even maladaptive (resulting in a maintained or worsened sad mood in children).

A mood-congruent gaze toward sad faces, has also previously been reported for individuals with an induced sad mood (Bradley et al., 1997; Joormann et al., 2007; Isaacowitz et al., 2008; Kujawa et al., 2011) and dysphoric adults (e.g., Bradley et al., 1997; Koster et al., 2005). In addition, a mood-congruent gaze away from negative stimuli was also reported (e.g., McCabe et al., 2000). The mood-congruent gaze away from facial stimuli was observed in healthy individuals (McCabe et al., 2000; Koster et al., 2005; Joormann and Gotlib, 2007) and was interpreted as a “protective bias” to avoid a sad mood (McCabe et al., 2000). This avoidance tendency was also found in children suffering from childhood adversity, namely from maternal criticism (Gibb et al., 2011) or parental distress (Grossheinrich et al., submitted). Additionally, an avoidance bias away from negative stimuli was also found repeatedly in offspring of depressive mothers (e.g., Boyd et al., 2006) and in juvenile MDD (Harrison and Gibb, 2015), which is probably based on a different neurocognitive mechanism in contrast to adult MDD. As one possible mechanism, among others (Gibb et al., 2016), enhanced reward seeking in adolescence, which is discussed to overshadow adolescent MDD (Trinkl et al., 2015), may help adolescents to disengage their attention away from negative stimuli. In the present experiment, we argue that we were not able to observe any protective bias to avoid sadness, as a sad mood was voluntarily induced and experimentally desired.

The attentional bias toward fearful faces (which did not survive alpha correction) might be explained by the mood induction procedure. In the present study we used film clips which might induce somewhat mixed emotional states. Specifically, for a sad mood induction we presented a film clip in which a lion cub (child) loses his father, potentially also prompting fear in addition to sadness.

From a methodical point of view, our findings demonstrate the importance of considering the age of the individuals depicting facial expressions, in future studies. While on the one hand the preference to use facial expressions depicting children is commendable (e.g., in empathy research involving children), our findings suggest that these design decisions may also impact on the obtainable results.

### Limitations

One limitation of the present study was related to the exclusion of four children who did not respond to our mood induction procedure. Of course, it would be advisable to test a larger sample of participants in a follow-up study to ensure more substantial statistical power, which in the present study was slightly compromised by these few participants who did not meet our selection criteria. While it is difficult to draw any statistical conclusions here, it appears noteworthy that all individuals excluded were male, which would be in line with previous findings suggesting that male children are less sensitive to sad mood induction procedures (von Leupoldt et al., 2007). Indeed, the tendency of females to exhibit stronger valence ratings than males do, has been shown for adults (Gross and Levenson, 1995), and a similar gender effect on valence ratings during unpleasant emotional stimulation has been reported in children, with girls rating aversive pictures as being more unpleasant than boys (Sharp et al., 2006). Therefore, it may be that such a gender difference, related to mood reactivity, is generally present in children before entering adolescence. Corresponding effects of gender on mood-congruent attention biases therefore clearly deserve to be studied more explicitly in future studies using a larger pool of male and female participants.

Another limitation of the present study was our decision against implementing a neutral mood induction condition, which could serve as a baseline. However, we decided to keep the experiment sufficiently short to ensure that the children’s attention would not diminish. More importantly, a neutral mood induction would be challenging to establish in our laboratory setting as the initial and general mood of our participants was rather positive. Therefore, any “neutral” mood induction would result in a worsening of participants’ mood. In this study, the sad mood induction could therefore only be investigated against the backdrop of the positive mood induction. As a result, we cannot exclude that an attentional bias toward sad adult faces might also be present while children are in a neutral mood. More importantly, we also cannot rule out a more general caregiver mood-congruent bias that is not only present for a sad mood, but also for a happy mood. Given that our study design only allows for contrasting sad and happy moods (instead of having a neutral baseline), the overall effect could also principally be ascribed to a mood-congruent bias toward happy adult faces when in a happy mood. This follows the fact that neutral faces were presented in every trial for the purpose of the ratio score calculation (see Isaacowitz et al., 2008). As a consequence, the positive mood induction and the happy facial expressions served as relevant control conditions in the present experiment. Future research should therefore focus on the question of whether the observed bias is mainly driven by a sad or happy mood-congruent bias, when compared to a neutral baseline, by using a different research design.

Note that we deliberately decided to closely follow a previously established research design and its corresponding experimental conditions and dependent variables (Isaacowitz et al., 2008). This also included the involvement of three types of negative facial expressions and one type of positive emotional facial expression. However, setting up a future study involving solely happy and sad faces would increase the amount of trials and the corresponding signal to noise ratio.

A final important limitation here was the absence of an adult control group. Given the caregiver framework of the current study, we assumed the attentional bias toward adult faces in contrast to faces depicting children would be smaller with increasing age of the participants. Thus in future studies, individuals with a larger age range (including adults and even old adults) should be examined.

## Conclusion and Perspectives

In sum, we observed a mood-congruent visual attention bias toward sad facial expressions when a sad (as opposed to happy) mood was induced in children. Importantly, this attention bias was most pronounced for adult faces, which can be interpreted in terms of adaptive information seeking behavior. In future studies, gender differences could additionally be examined to determine if the attentional bias toward adult faces is specific for primary caregivers (mostly the mother, sometimes the father or both parents) or if the attentional biases are directed to a similar model (assuming that girls exhibit a female adult attentional bias whereas boys predominantly look at male facial expressions). In addition, in future studies attentional biases could be investigated in the face of conflict, for example, following an emotionally confusing scene (Rohr et al., 2016).

From a methodological point of view, the results suggest that great care should be employed in designing research protocols whenever facial stimuli are involved, since the age of the depicted faces may influence the outcomes and limit theoretical conclusions. In addition, from a clinical point of view, the findings might have important implications for the development of specific mood-related interventions by parents, teachers and psychotherapists.

## Ethics Statement

The study was approved by the Ethics committee of the Medical Faculty of RWTH Aachen (EK 291/11).

## Author Contributions

NG, LH, MS-R, and AvL designed the research. NG, CF, and KK contributed to the interpretation of the data. NG conducted the experiments and analyzed the data. NG and LH wrote the paper. All authors approved the final version of the manuscript.

## Conflict of Interest Statement

The authors declare that the research was conducted in the absence of any commercial or financial relationships that could be construed as a potential conflict of interest.

## References

[B1] Achenbach T. M. (1991). *Manual for the Child Behavior Checklist/ 4-18 and 1991 Profile.* Burlington VT: Department of Psychiatry, University of Vermont.

[B2] Arbeitsgruppe Deutsche Child Behavior Checklist. (1998). *Elternfragebogen über das Verhalten von Kindern und Jugendlichen.Ddeutsche Bearbeitung der Child Behavior Checklist (CBCL/*4-18). Einführung und Anleitung zur Handauswertung mit Deutschen Normen. Köln: Arbeitsgruppe Kinder-, Jugend- und Familiendiagnostik (KJFD).

[B3] Beck A. T. (1967). *Depression: Clinical, Experimental, and Theoretical Aspects.* New York, NY: Harper and Row.

[B4] Beck A. T. (1976). *Cognitive Therapy and the Emotional Disorders.* New York, NY: New American Library.

[B5] Bowlby J. (1969). *Attachment and Loss: Attachment* Vol. 1 2nd Edn. New York, NY: Basic Books.

[B6] Bowlby J. (1988). *A Secure Base: Clinical Applications of Attachment Theory.* London: Routledge.

[B7] Boyd R. C. Zayas L. H. McKee M. D. (2006). Mother-infant interaction, life events and prenatal and postpartum depressive symptoms among urban minority women in primary care. *Matern. Child Health J.* 10 139–148. 10.1007/s10995-005-0042-2 16397831

[B8] Bradley B. P. Mogg K. Lee S. C. (1997). Attentional biases for negative information in induced and naturally occurring dysphoria. *Behav. Res. Ther.* 35 911–927. 10.1016/S0005-7967(97)00053-3 9401132

[B9] Bradley B. P. Mogg K. Millar N. White J. (1995). Selective processing of negative information: effects of clinical anxiety, concurrent depression, and awareness. *J. Abnorm. Psychol.* 104 532–536. 10.1037/0021-843X.104.3.532 7673577

[B10] Carstensen L. Mikels J. Mather M. (2006). “Aging and the intersection of cognition, motivation and emotion,” in *Handbook of the Psychology of Aging. 6*, eds Birren J. Schaie K. (San Diego, CA: Academic Press), 343–362.

[B11] Carstensen L. L. (2006). The influence of a sense of time on human development. *Science* 312 1913–1915. 10.1126/science.1127488 16809530 PMC2790864

[B12] Cheng P. Preston S. D. Jonides J. Mohr A. H. Thummala K. Casement M. (2015). Evidence against mood-congruent attentional bias in major depressive disorder. *Psychiatry Res.* 230 496–505. 10.1016/j.psychres.2015.09.043 26477954

[B13] Clasen P. C. Wells T. T. Ellis A. J. Beevers C. G. (2012). Attentional biases and the persistence of sad mood in major depressive disorder. *J. Abnorm. Psychol.* 122 74–85. 10.1037/a0029211 22867117 PMC3856951

[B14] Cloninger C. R. (1986). A unified biosocial theory of personality and its role in the development of anxiety states. *Psychiatr. Dev.* 4 167–226. 3809156

[B15] Ekman P. Friesen W. V. (1976). *Pictures of Facial Affect.* Palo Alto, CA: Consulting Psychologists Press.

[B16] Faul F. Erdfelder E. Lang A. G. Buchner A. (2007). G^∗^ Power 3: a flexible statistical power analysis program for the social, behavioral, and biomedical sciences. *Behav. Res. Methods* 39 175–191. 10.3758/BF03193146 17695343

[B17] Fritzsche A. Dahme B. Gotlib I. H. Joormann J. Magnussen H. Watz H. (2010). Specificity of cognitive biases in patients with current depression and remitted depression and in patients with asthma. *Psychol. Med.* 40 815–826. 10.1017/S0033291709990948 19719897 PMC2847035

[B18] Gibb B. E. Johnson A. L. Benas J. S. Uhrlass D. J. Knopik V. S. McGeary J. E. (2011). Children’s 5-HTTLPR genotype moderates the link between maternal criticism and attentional biases specifically for facial displays of anger. *Cogn. Emot.* 25 1104–1120. 10.1080/02699931.2010.508267 21895572 PMC3170093

[B19] Gibb B. E. Mcgeary J. E. Beevers C. G. (2016). Attentional biases to emotional stimuli: key components of the RDoC constructs of sustained threat and loss. *Am. J. Med. Genet. Part B* 171 65–80. 10.1002/ajmg.b.32383 26369836 PMC5664953

[B20] Goth K. Schmeck K. (2009). *JTCI-Das Junior Temperament und Charakter Inventar. Eine Inventarfamilie zur Erfassung der Persönlichkeit vom Kindergarten- bis zum Jugendalter nach Cloningers Biopsychosozialem Persönlichkeitsmodell.* Göttingen: Hogrefe.

[B21] Gotlib I. H. Cane D. B. (1987). Construct accessibility and clinical depression: a longitudinal investigation. *J. Abnorm. Psychol.* 96 199–204. 10.1037/0021-843X.96.3.199 3680757

[B22] Gotlib I. H. Kasch K. L. Traill S. Joormann J. Arnow B. A. Johnson S. L. (2004). Coherence and specificity of information-processing biases in depression and social phobia. *J. Abnorm. Psychol.* 113 386–398. 10.1037/0021-843X.113.3.386 15311984

[B23] Gotlib I. H. McCann C. D. (1984). Construct accessibility and depression: an examination of cognitive and affective factors. *J. Pers. Soc. Psychol.* 47 427–439. 10.1037/0022-3514.47.2.427 6481620

[B24] Gross J. J. Levenson R. W. (1995). Emotion elicitation using films. *Cogn. Emot.* 9 87–108. 10.1080/02699939508408966

[B25] Haaga D. A. Dyck M. J. Ernst D. (1991). Empirical status of cognitive theory of depression. *Psychol. Bull.* 110 215–236. 10.1037/0033-2909.110.2.215 1946867

[B26] Hansenne M. Reggers J. Pinto E. Kjiri K. Ajamier A. Ansseau M. (1999). Temperament and character inventory (TCI) and depression. *J. Psychiatr. Res.* 33 31–36. 10.1016/S0022-3956(98)00036-3 10094237

[B27] Harrison A. J. Gibb B. E. (2015). Attentional biases in currently depressed children: an eye-tracking study of biases in sustained attention to emotional stimuli. *J. Clin. Child Adolesc. Psychol.* 44 1008–1014. 10.1080/15374416.2014.930688 25010145 PMC4289475

[B28] Hodes R. L. Cook E. W. Lang P. J. (1985). Individual differences in autonomic response: conditioned association or conditioned fear? *Psychophysiology* 22 545–560. 10.1111/j.1469-8986.1985.tb01649.x 4048355

[B29] Isaacowitz D. M. Toner K. Goren D. Wilson H. R. (2008). Looking while unhappy: mood-congruent gaze in young adults, positive gaze in older adults. *Psychol. Sci.* 19 848–853. 10.1111/j.1467-9280.2008.02167.x 18947348 PMC2760922

[B30] Joormann J. Gotlib I. H. (2006). Is this happiness i see? Biases in the identification of emotional facial expressions in depression and social phobia. *J. Abnorm. Psychol.* 115 705–714. 10.1037/0021-843X.115.4.705 17100528

[B31] Joormann J. Gotlib I. H. (2007). Selective attention to emotional faces following recovery from depression. *J. Abnorm. Psychol.* 116 80–85. 10.1037/0021-843X.116.1.80 17324018

[B32] Joormann J. Talbot L. Gotlib I. H. (2007). Biased processing of emotional information in girls at risk for depression. *J. Abnorm. Psychol.* 116 135–143. 10.1037/0021-843X.116.1.135 17324024

[B33] Koster E. H. W. De Raedt R. Goeleven E. Franck E. Crombez G. (2005). Mood-congruent attentional bias in dysphoria: maintained attention to and impaired disengagement from negative information. *Emotion* 5 446–455. 10.1037/1528-3542.5.4.446 16366748

[B34] Kujawa A. J. Torpey D. Kim J. Hajcak G. Rose S. Gotlib I. H. (2011). Attentional biases for emotional faces in young children of mothers with chronic or recurrent depression. *J. Abnorm. Child Psychol.* 39 125–135. 10.1007/s10802-010-9438-6 20644991 PMC3367881

[B35] Langner O. Dotsch R. Bijlstra G. Wigboldus D. H. J. Hawk S. T. van Knippenberg A. (2010). Presentation and validation of the radboud faces database. *Cogn. Emot.* 24 1377–1388. 10.1080/02699930903485076

[B36] Lewinsohn P. M. Steinmetz J. L. Larson D. W. Franklin J. (1981). Depression-related cognitions: antecedent or consequence? *J. Abnorm. Psychol.* 90 213–219. 10.1037/0021-843X.90.3.213 7288016

[B37] Luby J. L. Svrakic D. M. McCallum K. Przybeck T. R. Cloninger C. R. (1999). The junior temperament and character inventory: preliminary validation of a child self-report measure. *Psychol. Rep.* 84(3 Pt 2), 1127–1138. 10.2466/pr0.1999.84.3c.1127 10477935

[B38] Mancini G. Agnoli S. Baldaro B. Ricci Bitti P. E. Surcinelli P. (2013). Facial expressions of emotions: recognition accuracy and affective reactions during late childhood. *J. Psychol.* 147 599–617. 10.1080/00223980.2012.727891 24199514

[B39] McCabe S. B. Gotlib I. H. Martin R. A. (2000). Cognitive vulnerability for depression: deployment of attention as a function of history of depression and current mood state. *Cogn. Ther. Res.* 24 427–444. 10.1023/A:1005579719849

[B40] Mogg K. Bradley B. P. Hallowell N. (1994). Attentional bias to threat: roles of trait anxiety, stressful events, and awareness. *Q. J. Exp. Psychol. A* 47 841–864. 10.1080/14640749408401099 7809399

[B41] Mogg K. Bradley B. P. Williams R. (1995). Attentional bias in anxiety and depression: the role of awareness. *Br. J. Clin. Psychol.* 34 17–36. 10.1111/j.2044-8260.1995.tb01434.x 7757037

[B42] Mogg K. Bradley B. P. Williams R. Mathews A. (1993). Subliminal processing of emotional information in anxiety and depression. *J. Abnorm. Psychol.* 102 304–311. 10.1037/0021-843X.102.2.304 8315143

[B43] Mogg K. Waters A. M. Bradley B. P. (2017). Attention bias modification (ABM): review of effects of multisession ABM training on anxiety and threat-related attention in high-anxious individuals. *Clin. Psychol. Sci.* 5 698–717. 10.1177/2167702617696359 28752017 PMC5513441

[B44] Okon-Singer H. (2018). The role of attention bias to threat in anxiety: mechanisms, modulators and open questions. *Curr. Opin. Behav. Sci.* 19 26–30. 10.1016/j.cobeha.2017.09.008

[B45] Picci G. Scherf K. S. (2016). From caregivers to peers: puberty shapes human face perception. *Psychol. Sci.* 27 1461–1473. 10.1177/0956797616663142 27658903

[B46] Rinck M. Becker E. S. (2005). A comparison of attentional biases and memory biases in women with social phobia and major depression. *J. Abnorm. Psychol.* 114 62–74. 10.1037/0021-843X.114.1.62 15709813

[B47] Rohr C. S. Villringer A. Solms-Baruth C. van der Meer E. Margulies D. S. Okon-Singer H. (2016). The neural networks of subjectively evaluated emotional conflicts. *Hum. Brain Mapp.* 37 2234–2246. 10.1002/hbm.23169 26991156 PMC6867502

[B48] Scherf K. S. Scott L. S. (2012). Connecting developmental trajectories: biases in face processing from infancy to adulthood. *Dev. Psychobiol.* 54 643–663. 10.1002/dev.21013 22711622

[B49] Sharp C. van Goozen S. Goodyer I. (2006). Children’s subjective emotional reactivity to affective pictures: gender differences and their antisocial correlates in an unselected sample of 7-11-year-olds. *J. Child Psychol. Psychiatry* 47 143–150. 10.1111/j.1469-7610.2005.01464.x 16423145

[B50] Silk J. S. Steinberg L. Morris A. S. (2003). Adolescents’ emotion regulation in daily life: links to depressive symptoms and problem behavior. *Child Dev.* 74 1869–1880. 10.1046/j.1467-8624.2003.00643.x 14669901

[B51] Smith D. J. Duffy L. Stewart M. E. Muir W. J. Blackwood D. H. R. (2005). High harm avoidance and low self-directedness in euthymic young adults with recurrent, early-onset depression. *J. Affect. Disord.* 87 83–89. 10.1016/j.jad.2005.03.014 15967233

[B52] Teasdale J. D. (1983). Negative thinking in depression: cause, effect, or reciprocal relationship? *Adv. Behav. Res. Ther.* 5 3–25. 10.1016/0146-6402(83)90013-9

[B53] Trinkl M. Greimel E. Bartling J. Grünewald B. Schulte-Körne G. Grossheinrich N. (2015). Right-lateralization of N2-amplitudes in depressive adolescents: an emotional go/no-go study. *J. Child Psychol. Psychiatry* 56 76–86. 10.1111/jcpp.12282 24963551

[B54] Unruh K. E. Bodfish J. W. Gotham K. O. (2018). Adults with autism and adults with depression show similar attentional biases to social-affective images. *J. Autism. Dev. Disord.* 10.1007/s10803-018-3627-5 [Epub ahead of print]. 29882107 PMC6286233

[B55] von Leupoldt A. Rohde J. Beregova A. Thordsen-Sörensen I. zur Nieden J. Dahme B. (2007). Films for eliciting emotional states in children. *Behav. Res. Methods* 39 606–609. 10.3758/BF03193032 17958174

[B56] Waters H. S. Rodrigues L. M. Ridgeway D. (1998). Cognitive underpinnings of narrative attachment assessment. *J. Exp. Child Psychol.* 71 211–234. 10.1006/jecp.1998.2473 9878106

[B57] Zvielli A. Bernstein A. Koster E. H. (2015). Temporal dynamics of attentional bias. *Clin. Psychol. Sci.* 3 772–788. 10.1177/2167702614551572

